# Theoretical Study of the Phonon and Electrical Conductivity Properties of Pure and Sr-Doped LaMnO_3_ Thin Films

**DOI:** 10.3390/ma17091995

**Published:** 2024-04-25

**Authors:** Angel T. Apostolov, Iliana N. Apostolova, Julia Mihailowa Wesselinowa

**Affiliations:** 1 University of Architecture, Civil Engineering and Geodesy, 1046 Sofia, Bulgaria; angelapos@abv.bg; 2 University of Forestry, 1756 Sofia, Bulgaria; inaapos@abv.bg; 3 Faculty of Physics, Sofia University “St. Kliment Ohridski”, J. Bouchier Blvd. 5, 1164 Sofia, Bulgaria

**Keywords:** Sr-doped LaMnO_3_ thin films, phonon energy and damping, electrical conductivity, microscopic model, Green’s function theory

## Abstract

The film thickness, temperature, substrate and doping dependence of the phonon energy ω and damping γ, as well as the electrical conductivity, of pure and Sr-doped LaMnO_3_ thin films near the phase transition temperature $T_{N}$ are investigated using a microscopic model and the Green’s function technique. Due to the strong spin–phonon interaction, there appears a kink at $T_{N}$ in the temperature dependence of ω(T) and γ(T). The softening and hardening of the ω = 495 cm^−1^ (A_1*g*_) and ω = 614 cm^−1^ (B_2*g*_) modes is explained by the different sign of the anharmonic spin–phonon interaction constant *R*. The damping increases with *T* for both cases because it is proportional to $R^{2}$. ω decreases whereas γ increases with an increasing Sr concentration. This is due to the strain caused by the difference between the ionic radii of the La and Sr ions. The film thickness dependence is also considered. ω and γ increase strongly with the decreasing film thickness. The electrical conductivity is enhanced after the doping of the LMO thin films with Sr, which could be used for energy storage applications. The observed results are in good qualitative agreement with the experimental data.

## 1. Introduction

Ion-doped manganites with the general formula *R*_1−*x*_A_*x*_MnO_3_ (*R* is a rare-earth element; A = Ca, Sr, Ba or Pb) and a perovskite structure possess interesting physical properties. Recently, Koriba et al. [1] have used density functional theory (DFT) in order to study the structural, electronic, magnetic and mechanical properties of LaMnO_3_ (LMO) in its orthorhombic, cubic and rhombohedral phases. The properties of LMO have been studied intensively in the last few years since it was found that the partial substitution of La by Ca, Sr or Ba results in structural changes and the occurrence of colossal magnetoresistance near the temperatures of the spin ordering of Mn ions [2]. Hess et al. [3] have studied 20% (Ca, Sr, Ba)-doped LMO using a DFT-based defect chemistry model. Sr substitution at the A-site of LMO nanoparticles could be used for energy applications because it shows a higher current value and higher conductivity, as described by Gupta et al. [4].

LMO undergoes a phase transition from the low-temperature A-type antiferromagnetic phase to the paramagnetic phase at ∼140 K and to the orbital disordered phase above 780 K. Let us emphasize that LMO thin films show ferromagnetic properties [5]. It must also be mentioned that antiferromagnetic nanostructures exhibit large magnetization and coercive fields below the phase transition temperature $T_{N}$ compared to their bulk materials, which is due to the uncompensated surface spins [6]. By doping with Sr, bulk LMO becomes also ferromagnetic [7].

Although it is expected that structural changes and magnetic ordering will also influence the phonon spectra in Sr-doped LMO, these have not been sufficiently theoretically investigated. The changes in the lattice parameters with temperature manifest in a shift in the line position and intensity, as well as in the line width of the Raman peaks. Moreover, Raman spectroscopy is a useful method to study phase transitions. At 80 K, the lines of the $A_{g}$ symmetry are observed at 148, 210, 267, 295, 452 and 495 cm^−1^ and those of the $B_{1g}$ symmetry at 197, 313, 436 and 606 cm^−1^ [8]. The Raman spectra of Sr-doped bulk LMO manganites are studied in [9,10,11], whereas those of pure and Sr-doped LMO thin films and nanoparticles are investigated in [12,13,14,15,16,17,18]. Recently, Helton et al. [19] have studied the damping and softening of transverse acoustic phonons in La_0.7_Sr_0.3_MnO_3_. The doping dependence of the phonon frequencies ω in Sr-doped LMO has been investigated experimentally by many authors [10,11,20,21]. It is observed that ω decreases when increasing the Sr concentration.

It must be mentioned that most theoretical works consider the magnetic properties of LMO. Wdowik et al. [22] calculated the vibrational dynamics of the undoped LMO from first principles by DFT. Talati et al. [23] and Rini et al. [24] investigated the phonon dispersion curves of LMO by using a lattice dynamical simulation method and an interatomic shell model potential, respectively.

The aim of the present paper is to use a microscopic model and the Green’s function theory to study the phonon energy and damping, as well as the electrical conductivity, of Sr-doped LMO thin films in dependence on the film thickness, temperature, substrate and doping concentration. To our knowledge, such studies have not yet been performed using this method or density functional theory.

## 2. The Model

LMO, in which Mn^3+^ is the only present high-spin magnetic ion with *S* = 2, is an antiferromagnetic compound (A-type) whose Neel temperature is $T_{N}$ = 140 K. It shows also orbital ordering (C-type) below 780 K, which will be not considered here. The Jahn–Teller electronic ordering couples the Mn^3+^ spins within the basal planes with ferromagnetic coupling (superexchange interaction). These planes are coupled one to another with antiferromagnetic coupling. The magnetic properties of ion-doped LMO with doping concentration *x* are described by the Heisenberg Hamiltonian:

(1) $$H_{sp}=-\frac{1}{2}\sum_{i,j}\left(1-x\right)J_{ij}S_{i}\cdot S_{j}-\frac{1}{2}\sum_{i,j}xJ_{dij}S_{i}\cdot S_{j}-D_{z}\sum_{i}{\left(S_{i}^{z}\right)}^{2},$$

where $S_{i}$ and its *z* component $S_{i}^{z}$ are spin operators for the localized Mn^3+^ spins at site *i*. $J_{ij}$ stands for the spin exchange interaction between the nearest neighboring Mn ions in the planes ($J_{1}>0$) and for the exchange coupling between the next nearest neighboring Mn ions between these planes ($J_{2}<0$). $J_{dij}$ is the exchange interaction constant for the doped case, which, due to the different ionic radii between the Sr and La ions, can be changed compared to the undoped case $J_{ij}$. $D_{z}>0$ is the single-site anisotropy parameter of the easy-axis type.

A strong spin–phonon interaction in LMO is reported [9,11], which must be taken into account in order to obtain the correct results:

(2) $$H^{sp-ph}=-\frac{1}{2}\sum_{i,j,k}F\left(i,j,k\right)Q_{i}S_{j}^{z}S_{k}^{z}-\frac{1}{4}\sum_{i,j,r,s}R\left(i,j,r,s\right)Q_{i}Q_{j}S_{r}^{z}S_{s}^{z}+h.c.$$

The normal coordinate $Q_{i}$ can be expressed in terms of phonon creation $a^{+}$ and annihilation *a* operators $Q_{i}={\left(2\omega_{0i}\right)}^{-1/2}\left(a_{i}+a_{i}^{+}\right)$. $\omega_{0i}$ is the frequency of the lattice mode. *F* and *R* are the spin–phonon coupling constants in the first and second order, respectively. The spin–phonon interaction renormalizes the spin exchange interaction constant *J* to $J_{eff}=J+2F^{2}/\left(\omega_{o}-MR\right)$. *M* is the magnetization.

$H_{ph}$ contains the lattice vibrations, including anharmonic phonon–phonon interactions:

(3) $$\begin{array}{ccc} H_{ph} & = & \frac{1}{2!}\sum_{i}\omega_{0i}a_{i}^{+}a_{i}+\frac{1}{3!}\sum_{i,j,r}B\left(i,j,r\right)Q_{i}Q_{j}Q_{r}\\ & + & \frac{1}{4!}\sum_{i,j,r,s}A\left(i,j,r,s\right)Q_{i}Q_{j}Q_{r}Q_{s}. \end{array}$$

*A* and *B* are three-phonon and four-phonon anharmonic interaction constants, respectively.

For the approximate calculation of the Green’s functions, we use the method proposed by Tserkovnikov [25]. It goes beyond random phase approximation, taking into account all correlation functions. Moreover, this method allows us to calculate the imaginary part of the Green’s function. We wish now to sketch it briefly. After the formal integration of the equation of motion for the retarding two-time Green’s function

(4) $$G_{ij}\left(t\right)=\langle \langle a_{i}\left(t\right);a_{j}^{+}\rangle \rangle$$

one obtains

(5) $$G_{ij}\left(t\right)=-i\theta \left(t\right)\langle \left[a_{i};a_{j}^{+}\right]\rangle \exp\left(-i\omega_{ij}\left(t\right)t\right),$$

with θ(x) = 1 for x>0, θ(x) = 0 for x<0,

(6) $$\begin{array}{ccc} \omega_{ij}\left(t\right)=\omega_{ij} & - & \frac{i}{t}\int_{0}^{t}dt^{\prime }t^{\prime }\left(\frac{\langle \left[j_{i}\left(t\right);j_{j}^{+}\left(t^{\prime }\right)\right]\rangle }{\langle \left[a_{i}\left(t\right);a_{j}^{+}\left(t^{\prime }\right)\right]\rangle }\right.\\ & - & \left.\frac{\langle \left[j_{i}\left(t\right);a_{j}^{+}\left(t^{\prime }\right)\right]\rangle \langle \left[a_{i}\left(t\right);j_{j}^{+}\left(t^{\prime }\right)\right]\rangle }{{\langle \left[a_{i}\left(t\right);a_{j}^{+}\left(t^{\prime }\right)\right]\rangle }^{2}}\right) \end{array}$$

and $j_{i}\left(t\right)=\langle \left[a_{i},H_{interaction}\right]\rangle$. The time-independent term

(7) $$\omega_{ij}=\frac{\langle \left[\left[a_{i},H\right];a_{j}^{+}\right]\rangle }{\langle \left[a_{i};a_{j}^{+}\right]\rangle }$$

is the excitation energy in the generalized Hartree–Fock approximation. The time-dependent term in Equation (7) includes damping effects.

We obtain from the full Hamiltonian the following expression for the phonon energy, which is renormalized through the spin–phonon interactions:

(8) $$\omega_{ij}^{2}=\omega_{0}^{2}-2\omega_{0}\left(M_{i}M_{j}R_{ij}\delta_{ij}-\frac{1}{2N}\sum_{r}A_{ijr}^{ph}\left(2{\bar{N}}_{r}+1\right)-B_{ij}^{ph}\langle Q_{ij}\rangle \delta_{ij}\right),$$

with

(9) $$\langle Q_{ij}\rangle =\frac{M_{i}M_{j}F_{ij}\delta_{ij}-\frac{1}{N^{\prime }}\sum_{r}B_{ijr}^{ph}\left(2{\bar{N}}_{r}+1\right)}{\omega_{0}-M_{i}M_{j}R_{ij}\delta_{ij}+\frac{1}{N^{\prime }}\sum_{r}A_{ijr}^{ph}\left(2{\bar{N}}_{r}+1\right)}.$$

The correlation function of the phonons ${\bar{N}}_{i}=\langle a_{i}^{+}a_{i}^{-}\rangle$ is obtained via the spectral theorem. $M_{i}$ is the magnetization, which is calculated from the Green’s function $\langle \langle S_{i}^{+};S_{j}^{-}\rangle \rangle$. We obtain, for an arbitrary spin *S* value,

(10) $$M=\langle S^{z}\rangle =\frac{1}{N}\sum_{i}\left[\left(S+0.5\right)\coth\left[\left(S+0.5\right)\beta E_{i}\right]-0.5\coth\left(0.5\beta E_{i}\right)\right].$$

$E_{i}$ are the spin wave energies, $\beta =1/k_{B}T$.

The phonon damping is also calculated taking into account the anharmonic spin–phonon and phonon–phonon interactions:

(11) $$\gamma =\gamma_{sp-ph}+\gamma_{ph-ph}.$$

$\gamma_{sp-ph}$ is the damping due to the spin–phonon interaction:

(12) $$\begin{array}{ccc} \gamma_{sp-ph}\left(rs\right) & = & \frac{2\pi }{N^{2}}\sum_{ij}F_{ijr}^{2}\langle S_{i}^{z}\rangle \langle S_{j}^{z}\rangle \left({\bar{n}}_{i}-{\bar{n}}_{j}\right)\delta_{rs}\delta \left(E_{i}-E_{j}-\omega_{r}\right)\\ & + & \frac{2\pi }{N^{3}}\sum_{ijl}R_{ijlr}^{2}\left({\langle S_{i}^{z}\rangle }^{2}{\langle S_{j}^{z}\rangle }^{2}\delta_{rs}+\left[\left({\bar{n}}_{i}-{\bar{n}}_{j}\right)\left(1+{\bar{N}}_{l}\right)+{\bar{n}}_{i}\left(1+{\bar{n}}_{j}\right)\right]\right)\\ & * & \delta (E_{i}-E_{j}-\omega_{l}+\omega_{r}). \end{array}$$

$\gamma_{ph-ph}$ is the damping part that arises from the phonon–phonon interaction:

(13) $$\begin{array}{ccc} \gamma_{ph-ph}\left(rs\right) & = & \frac{3\pi }{N^{2}}\sum_{ij}B_{ijr}^{2}\left({\bar{N}}_{i}-{\bar{N}}_{j}\right)\delta_{rs}\left[\delta \left(-\omega_{i}-\omega_{j}+\omega_{r}\right)-\delta \left(\omega_{i}-\omega_{j}+\omega_{r}\right)\right]\\ & + & \frac{4\pi }{N^{3}}\sum_{ijl}A_{ijlr}^{2}\delta_{rs}\left[{\bar{N}}_{i}\left(1+{\bar{N}}_{j}+{\bar{N}}_{l}\right)-{\bar{N}}_{j}{\bar{N}}_{l}\right]\\ & * & \delta (\omega_{i}-\omega_{j}+\omega_{l}-\omega_{r}). \end{array}$$

$E_{i}$ and $\omega_{i}$ are the magnetic and phonon energies. The correlation functions ${\bar{n}}_{i}=\langle S_{i}^{-}S_{i}^{+}\rangle$ and ${\bar{N}}_{i}=\langle a_{i}^{+}a_{i}^{-}\rangle$ are obtained via the spectral theorem. It must be mentioned that in the damping γ at low temperatures, the terms due to the anharmonic spin–phonon interaction play an important role, whereas, at higher temperatures, above the phase transition temperature, only the anharmonic phonon–phonon terms remain.

In order to calculate the electrical conductivity σ, we need the Hubbard model:

(14) $$H=\sum_{ij\sigma }t_{ij}c_{i\sigma }^{+}c_{j\sigma }+U\sum_{i}n_{i↑}n_{i↓},$$

where $t_{ij}$ is the hopping integral, *U* is the Coulomb repulsion, $n_{i\sigma }=c_{i\sigma }^{+}c_{i\sigma }$ and $c_{i\sigma }^{+}$ and $c_{i\sigma }$ are Fermi operators.

The electrical conductivity σ can be observed from the following equation:

(15) $$\sigma^{\mu \nu }\left(\omega \right)-\frac{ie}{V}lim_{\delta \to 0^{+}}\sum_{ij\sigma }t_{ij}\left(R_{i}^{\mu }-R_{j}^{\mu }\right)\ll c_{i\sigma }^{+}c_{j\sigma };P^{\nu }\gg_{\omega +i\delta },$$

with $P^{\nu }=-e\sum i\sigma R_{i}^{\nu }n_{i\sigma }$; *e* is the electron charge and *V* is the volume.

We consider the Green’s function $G_{ij\sigma }\left(\omega \right)=\;\ll c_{i\sigma }^{+}c_{j\sigma };P^{\nu }\gg$, which can be written with the components

(16) $$G_{ij\sigma }\left(\omega \right)=\sum_{\alpha \beta }G_{ij\sigma }^{\alpha \beta }\left(\omega \right)=\sum_{\alpha \beta }\ll c_{i\sigma }^{+}c_{j\sigma }n_{i-\sigma }^{\alpha }n_{j-\sigma }^{\beta };P^{\nu }\gg .$$

For $G_{ij\sigma }^{\alpha \beta }\left(\omega \right)$, we obtain

(17) $$G_{ij\sigma }^{\alpha \beta }\left(\omega \right)=\frac{e\left(R_{j}^{\nu }-R_{i}^{\nu }\right)<c_{i\sigma }^{+}c_{j\sigma }n_{i-\sigma }^{\alpha }n_{j-\sigma }^{\beta }>}{\omega +U(\delta_{\alpha +}-\delta_{\beta +})}.$$

After the calculation of the correlation function using the spectral theorem, from Equations (12) and (13), we can observe the electrical conductivity σ.

## 3. Numerical Results and Discussion

The numerical calculations are performed in the JAVA programming environment using simple iterative procedures and summation over nearest neighbors. They are performed using the following model parameters: $J_{1}$ = 9.6 K, $J_{2}$ = −6.7 K, $D_{z}$ = 1.92 K [26], *S* = 2, *F* = 23 cm^−1^, *R* = −18 cm^−1^, *B* = −2.54 cm^−1^, *A* = 6.61 cm^−1^, *t* = 1 eV, *U* = 2 eV. The phonon–phonon interaction constants *A* and *B* are determined from the Raman spectra for temperatures above the Curie temperature $T_{C}$ (where the terms with *R* and *F* vanish), whereas the spin–phonon interaction constants *F* and *R*—arise at very low temperatures—taking two values at two different temperatures from the Raman phonon energy and solving the system of two equations with two unknown parameters.

The exchange interaction constant *J* depends on the distance between the spins, i.e., on the lattice parameter, on the different strains, on the lattice symmetry and on the number of next neighbors. It is inversely proportional to the distance between two neighboring spins. The ionic radius of the doping ion Sr^2+^ is r(Sr^2+^) = 1.44 $\dot{A}$, which is different compared to the host ion, r(La^3+^) = 1.36 $\dot{A}$. This means that, in this case, where r(Sr) > r(La), there appears a tensile strain. For the exchange interaction constant in the doped case $J_{d}$, we use the relation $J_{d}<J$. An increase in the lattice parameters and the cell volume was observed for Sr-doped LMO by Zheng et al. [27]. The doping concentration is taken into account by the factor (1−x) in Equation (1). For *x* = 0, without doping ions, *J* is the same as in the undoped compound and is maximal; then, with increasing *x*, *J* decreases, whereas $J_{d}$ increases. For *x* = 1, where all host ions are substituted with doping ions, the first term in Equation (1) vanishes. Only the second term with $J_{d}$ remains, which, for *x* = 0, is zero. This tensile strain, which reduces the exchange interaction constant, leads to a decrease in magnetization in Sr-doped LMO, in agreement with the experimental data of Wang et al. [28].

### 3.1. Film Thickness Dependence of the Phonon Energy and Damping of the A_1*g*_ Mode 495 cm^−1^ in Pure and Sr-Doped LMO Thin Films

**Figure 1:**
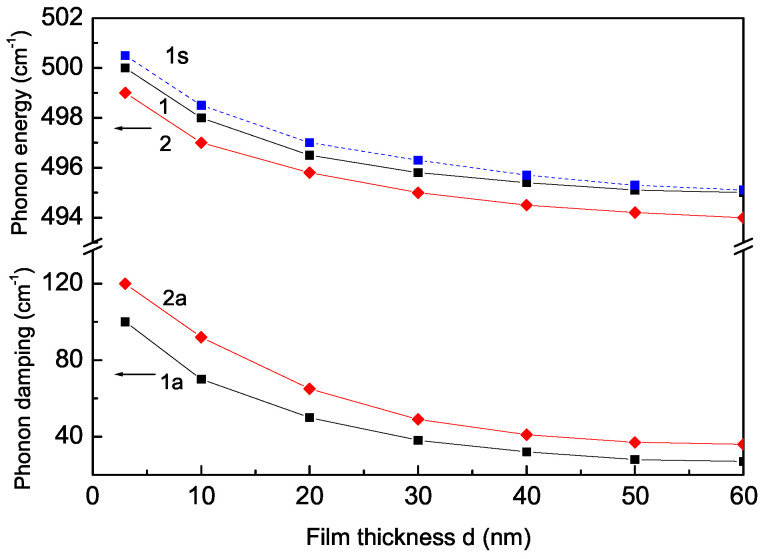
Film thickness dependence of the phonon energy of the A_1*g*_ mode ω = 495 cm^−1^ and their damping for *T* = 80 K in (1,1a) pure and (2,2a) Sr (*x* = 0.1) doped LMO thin film; (1s) LMO thin film on LaAlO_3_ substrate.

Firstly, we investigate the phonon energy and damping for the A_1*g*_ mode, ω = 495 cm^−1^, in pure and Sr-doped free LMO thin films. The film thickness dependence of the phonon mode for a pure (*x* = 0) LMO thin film is calculated. It must be noted that the oxygen excess in LMO films is compensated by a mixed valence state of the manganese cation (Mn^3+^/Mn^4+^). The exchange interaction constants on the two free surfaces (*n* = 1, *N*) $J_{s}$ are different from those in the bulk *J*; they can be larger or smaller. We have chosen the relation $J_{s}>J$, because Kharlamova et al. [29] have reported that, in LMO nanoparticles, the unit cell volume decreases. Then, through the spin–phonon interaction, we have $|R_{s}|>|R|$, R<0. The phonon energy ω, which corresponds to the Raman peak position, increases with the decreasing film thickness *d*, and the distance between the layers is taken to be around 1 nm (see Figure 1, curve 1). This means that the Raman mode positions shift towards a higher frequency compared to the bulk single crystal reported for pure LaMnO_3_ thin films [15]. The results show a remarkable difference in the phonon energies with the thickness. This behavior can be explained on the basis of the surface strain, which increases with decreasing thickness. As the thickness is increased, the phonon energies appear close to those of the single crystal.

Let us emphasize that the in-plane lattice parameters in LMO thin films are compressed, for example, by LaAlO_3_ and KTaO_3_ substrates [30]. This could be explained in our model by the exchange interaction constants between the film surface $J_{s}$ and the substrate $J_{ss}$, $J_{ss}>J_{s}$; the phonon energy is enhanced in comparison to the case without a substrate (see Figure 1, curve 1s). For substrates that lead to a tensile in-plane strain (for example, MgO [30,31]), we must use the relation $J_{ss}<J_{s}$. The phonon energy is reduced compared to the free thin film. The effects of the substrate-induced strain on the phonon modes in Sr-doped LMO thin films [12,13,14,15] will be considered in a future paper.

The film thickness dependence of the phonon energy ω(N) for a Sr-doped LMO thin film, *x* = 0.1, is also observed using the model parameters $J_{s}>J$, $|R_{s}|>|R|$, $J_{d}<J$ and $|R_{d}|<|R|$, R<0 (Figure 1, curve 2). The phonon energy ω increases again with the decreasing film thickness *d*, in agreement with the experimental data of Dore et al. [32]. The Sr doping could not change this behavior. However, it is observed that ω is smaller for the doped case (curve 2) compared to the undoped one (curve 1). This decreasing of ω for the Sr-doped LMO thin film is due to the different radii of the doped and host ions, due to the tensile strain, which appears after Sr doping in the LMO thin film. A similar decrease in ω with Sr is reported by many authors [10,11,20,21].

The phonon damping γ for pure and Sr-doped LMO thin films is also calculated. γ corresponds to the full width at half maximum (FWHM) of the Raman peaks. The results are shown in Figure 1. It can be seen that the phonon damping γ increases with the decreasing film thickness *d*. This means that the line widths of the Raman peaks also increase with the decreasing film thickness. Let us emphasize that γ is larger for the Sr-doped LMO thin film (curve 2a) than the undoped one (curve 1a). This is so because the doping contribution is additive to the surface one in the film, i.e.,

(18) $$\gamma =\gamma_{bulk}+\gamma_{surface}+\gamma_{doping}+\ldots$$

The broadening of the Raman peaks is reported after decreasing the film thickness in pure [15] and Sr-doped [32] LMO thin films. Let us emphasize that the distortion caused by the motions of oxygen atoms in Mn-O6 octahedra around the Mn ion are responsible for the Raman active vibrations. In doped manganites, the intensity and width of the broad bands are related to the amplitude of the dynamic fluctuations. The width of the instantaneous distribution of the Mn-O distances is the origin of the width of the Raman peaks. Therefore, as the Sr content increases, the activation energy decreases as well as the amplitude of the dynamic distortions. The width of the peaks corresponding to the *R*MnO_3_-type spectrum increases as the Sr content rises. Moreover, from a structural point of view, the doping by substitution violates the translational invariance, which naturally causes an increase in the scattering of the phonon modes, i.e., the damping, of the FWHM.

### 3.2. Temperature Dependence of the Phonon Energy and Damping of the A_1*g*_ Mode 495 cm^−1^ in Pure and Sr-Doped LMO Thin Films

**Figure 2:**
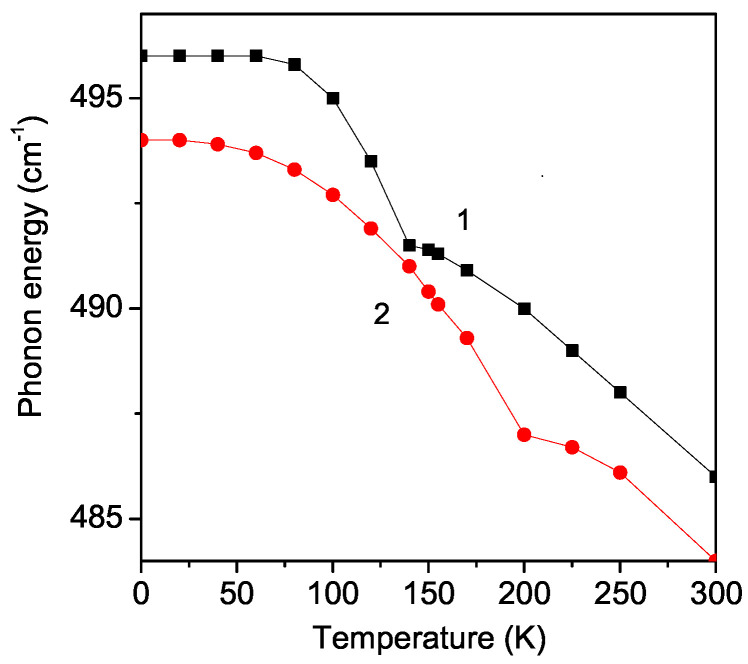
Temperature dependence of the phonon energy of the A_1*g*_ mode ω = 495 cm^−1^ in (1) pure and (2) Sr (*x* = 0.1) doped LMO thin film, *d* = 20 nm.

The temperature dependence of the phonon energy for the A_1*g*_ mode ω = 495 cm^−1^ in pure and Sr-doped LMO thin films is observed for Sr doping concentration *x* = 0.1 and using the following model parameters: $J_{s}>J$, $J_{d}<J$ and $|R_{d}|<|R|$, R<0. The results are presented in Figure 2. The phonon energy decreases with an increasing temperature. There is a kink at $T_{N}$ = 140 K in ω(T) for the pure LMO due to the spin–phonon interaction (curve 1). A similar kink was observed experimentally in pure LMO thin films by Dubey et al. [15] and Sr-doped bulk LMO by Bjoernsson et al. [21] and Podobedov et al. [10]. The kink in the Sr-doped LMO (which is already ferromagnetic) shifts to higher $T_{C}$ values. It can be seen from Figure 2 that, for *x* = 0.1, the kink is at $T_{C}$ = 200 K (curve 2). The increase in the Curie temperature of Sr-doped bulk LMO was reported also by Dimri et al. [33,34] and Ahmad et al. [35] using Raman and magnetic studies. The observed temperature behavior of Sr-doped LMO demonstrates the strong dependence of the phonons on both the temperature and the doping. The anomalies in the phonon energy are observed due to the effect of the ionic radius of the La/Sr site on both the doping and temperature. Thus, the results could be interpreted by considering the strong spin–phonon coupling in these compounds.

**Figure 3:**
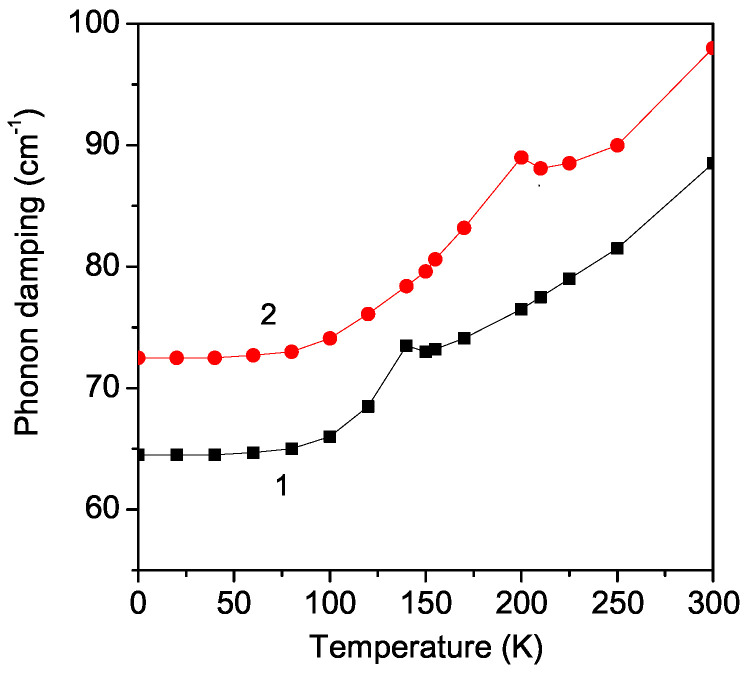
Temperature dependence of the phonon damping of the A_1*g*_ mode ω = 495 cm^−1^ in (1) pure and (2) Sr (*x* = 0.1) doped LMO thin film, *d* = 20 nm.

The phonon damping γ increases strongly with an increasing temperature *T*. This is supported by the experimentally observed broadening of the Raman peaks in these substances by Granado et al. [36]. There is an anomaly in the temperature dependence of the phonon damping γ of the A_1*g*_ mode ω = 495 cm^−1^ in the pure and Sr-doped LMO thin film around the phase transition temperatures: *T* = 140 K and *T* = 200 K for the undoped and Sr-doped LMO for *x* = 0.1 (see Figure 3). The anomalies in the phonon damping around the phase transition temperature $T_{N}$ in the pure and Sr-doped LMO are due to the strong anharmonic spin–phonon interaction *R*. It must be noted that $T_{N}$ is larger in the Sr-doped LMO than in the undoped one. The broadening of the Raman peaks in Sr-doped bulk LMO was reported by Podobedov et al. [10,37] and Choi et al. [11].

### 3.3. Temperature Dependence of the Phonon Energy and Damping of the B_2*g*_ Mode 614 cm^−1^ in Pure and Sr-Doped LMO Thin Films

**Figure 4:**
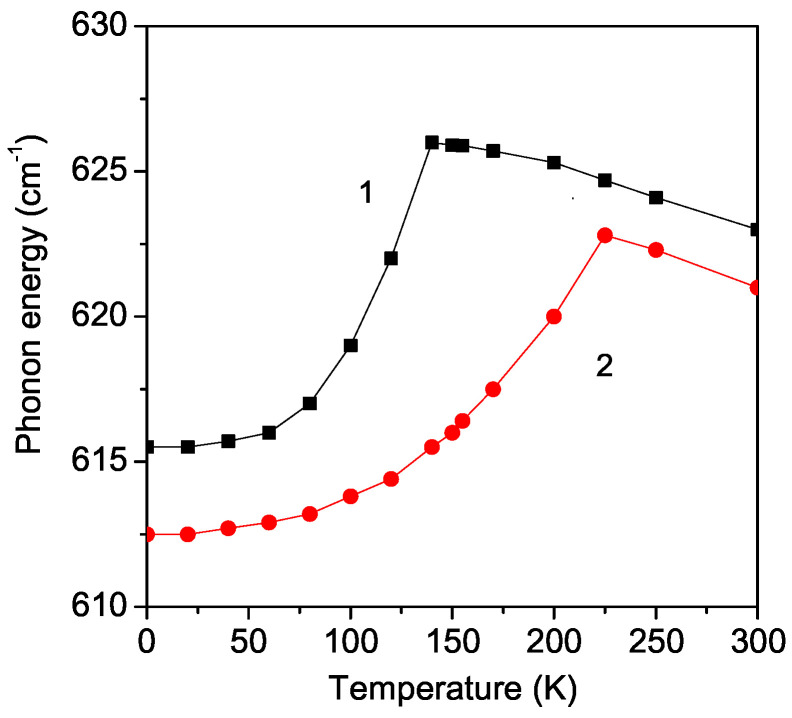
Temperature dependence of the phonon energy of the B_2*g*_ mode ω = 614 cm^−1^ in (1) pure and (2) Sr (*x* = 0.1) doped LMO thin film, *d* = 20 nm.

Now, we will investigate the phonon energy ω of the B_2*g*_ mode with ω = 614 cm^−1^. The experimental data of Dubey et al. [15] and Podobedov et al. [10] show an increase in ω with increasing temperature *T*. In order to obtain this hardening of ω, we choose a positive anharmonic spin–phonon constant R>0 [38], $J_{d}<J$ and $R_{d}<R$. The results are demonstrated in Figure 4. There is again a kink at the phase transition temperatures: *T* = 140 K for the undoped case and *T* = 225 K for the Sr-doped LMO thin film for *x* = 0.1. Moreover, above the kinks, and above the phase transition temperatures, the phonon energies ω decrease slightly with an increasing temperature. This is due to the fact that, above $T_{C}$, only the anharmonic phonon–phonon interactions *A* and *B* remain. The anharmonic spin–phonon interaction with negative R<0 and positive R>0 causes the different behavior of ω—softening or hardening below the phase transition temperature.

**Figure 5:**
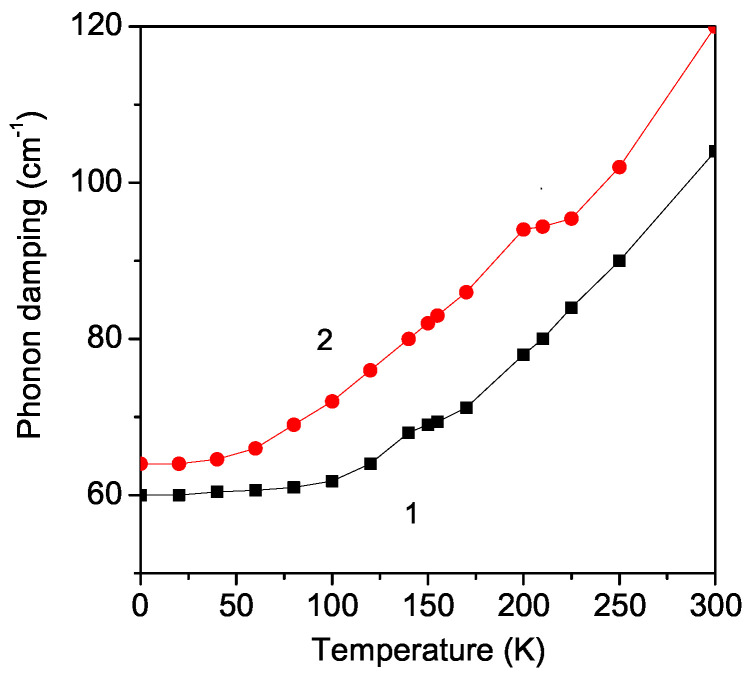
Temperature dependence of the phonon damping of the B_2*g*_ mode ω = 614 cm^−1^ in (1) pure and (2) Sr (*x* = 0.1) doped LMO thin film, *d* = 20 nm.

The phonon damping γ increases with temperature *T* (see Figure 5). There is again an anomaly around the phase transition temperatures: *T* = 140 K for the undoped case and *T* = 225 K for the Sr-doped LMO thin film for *x* = 0.1. It must be mentioned that γ is larger in the doped case compared to the undoped one for both cases—negative R<0 and positive R>0 (compare with Figure 4)—because the damping is proportional to $R^{2}$; see Equation (9). This behavior is due to the strong spin–phonon interaction *R*. An alternative means to observe the hardening of the phonon mode is the decrease in the Jahn–Teller distortion with increasing doping concentration *x* [13,36]. The reduction in the Jahn–Teller distortion in Sr-doped LMO has the same effect on the phonon energy as the change in the crystal symmetry due to the temperature-induced phase transition. This assumption is also confirmed by the polarized Raman spectra of doped crystals reported by Podobedov et al. [37]. Temperature-dependent Raman characterization has shown a metallic phase with a total reduction in the Jahn–Teller distortion in the rhombohedral La_0.67_Sr_0.33_MnO_3_ nanoparticles [18].

### 3.4. Sr Doping Effect in LMO Thin Films on the Electrical Conductivity

**Figure 6:**
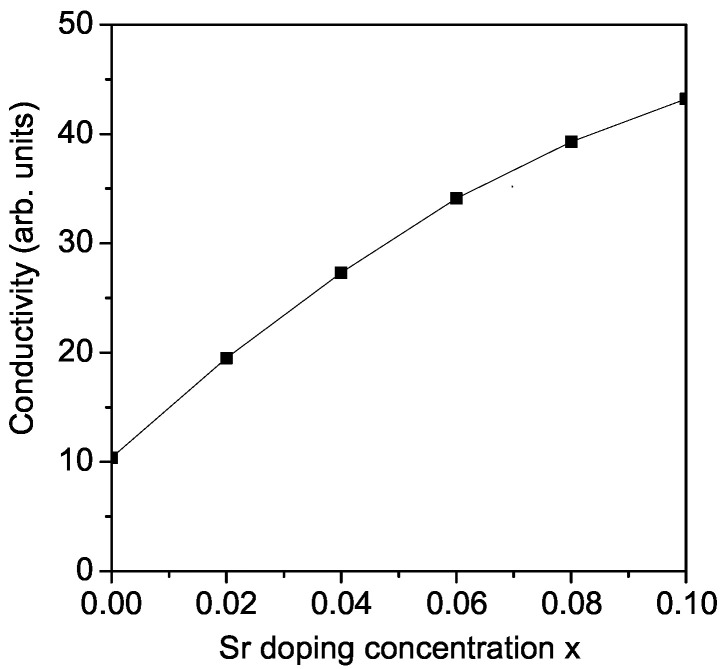
Sr doping concentration dependence of the electrical conductivity σ for LMO, *d* = 20 nm.

Among perovskites with an ABO_3_ structure, LMO is well known for solid oxide fuel cell (SOFC) applications. Strong electrical conductivity is necessary for the compound to serve as a cathode in SOFCs. Therefore, the doping of alkaline earth metals at the A-side enhances the electrical conductivity of the material [39]. Finally, from Equations (12)–(14), we have calculated the Sr concentration dependence of the electrical conductivity σ in an LMO thin film, *d* = 20 nm, *T* = 750 K. The substitution of La^3+^ with Sr^2+^ with a larger ionic radius favors both the crystal symmetry and the electrical conductivity of LMO. The results are presented in Figure 6. It can be seen that σ is enhanced when increasing the dopant concentration *x*; thus, it could be used for energy applications. This behavior is in qualitative agreement with the recently reported experimental results of Gupta et al. for Sr-doped LMO nanoparticles [4]. It must be noted that Gupta et al. [4] observed the highest σ increasing for *x* = 0.15. A similarly enhanced σ was obtained in Sr (on the La site) and Fe (on the Mn site) doped LMO by Shafi et al. [40] and transition metal-doped LaMn_*x*_$M_{\left(1-x\right)}$O_3_ (*M* = Fe, Co, Cr, Mn) [41]. Recently, He et al. [42] studied the electrical conductivity in Sr-doped La*M*O_3_ (*M* = Al, Ga, In, Er and Y) under varying oxygen partial pressures.

## 4. Conclusions

In conclusion, the film thickness, temperature, substrate and doping dependence of the phonon energy ω and damping γ of pure and Sr-doped LMO was investigated using a microscopic model and the Green’s function theory. The kink near $T_{N}$ = 140 K in pure LMO (due to strong spin–phonon interactions) shifts to higher *T* values with increasing Sr dopant. The softening and hardening of the ω = 495 cm^−1^ (A_1*g*_) and ω = 614 cm^−1^ (B_2*g*_) modes is explained by the different sign of the anharmonic spin–phonon interaction constant *R*, negative for the first case and positive for the second one. The damping increases with *T* for both cases, R<0 and R>0. In the temperature dependence of γ, there appears again an anomaly around the critical temperature. The phonon energy of the A_1*g*_ mode with ω = 295 cm^−1^ decreases, whereas the damping increases with an increasing Sr concentration. ω and γ increase strongly with the decreasing film thickness. The substrates can change also the phonon properties. The electrical conductivity is enhanced after the doping of LMO thin films with Sr, which could be used for energy storage applications.

Let us emphasize that the band gap is an important parameter in photocatalysts. Sr-doped LMO nanoparticles with low band gap energy of 2.2 eV could be used for energy applications [43]. The doping dependence of LMO with Sr and other doping ions will be investigated in a future paper.

## Data Availability

Data are contained within the article.
